# Perceptions of participation and the role of gender for the engagement in solar energy communities in Sweden

**DOI:** 10.1186/s13705-021-00312-6

**Published:** 2021-10-12

**Authors:** Daniela Lazoroska, Jenny Palm, Anna Bergek

**Affiliations:** 1 Institute for Urban Research, Malmö University, Nordenskiöldsgatan 1, 211 19 Malmö, Sweden; 2 International Institute for Industrial Environmental Economics, Lund University, Tegnérsplatsen 4, 221 00 Lund, Sweden; 3 Department of Technology Management and Economics, Chalmers University of Technology, 412 96 Gothenburg, Sweden

**Keywords:** Energy community, Gender, Participation, Decision-making, Energy justice, Solar energy, Sweden

## Abstract

**Background:**

Energy communities are emphasized by the EU as important for developing sustainable energy systems that include and engage many people. While many renewables are highly compatible with a more decentralized energy system, research indicates that participation in ‘desirable’ energy activities and energy decision-making is influenced by social and economic factors, including gender, economic status and home ownership. The overall aim of this article is to contribute to this line of inquiry by exploring how and under which conditions energy communities allow for broader participation in the energy system. This article examines how gender, as a more specific condition, influences the extent to which parties can or cannot engage with collective solar ownership models by means of a qualitative study of 11 solar energy communities and one housing association in Sweden.

**Results:**

The study revealed that despite the relative potential for inclusion that they hold, energy communities can raise justice concerns in terms of inequities concerning access, capacity, and opportunity to engage in decision-making.

**Conclusions:**

While solely focusing on gender offers a limited view of the dynamics of inclusion and exclusion in renewable energy projects, it is our position that integrating it into the analysis will provide insights into possible measures to remedy limitations and accelerate the renewable energy transition.

## Introduction and goals

The EU emphasizes energy communities as important for developing sustainable energy systems that include and engage many people. In *Clean Energy for all Europeans*, the EU Commission [1] emphasizes the potential of energy communities (or ‘community energy’) to develop an inclusive, equal, and efficient energy market (see also the Electricity Market Directive [2] and the Renewables Directive [3]). An energy community (hereon EC) is an umbrella term for different types of joint ownership of energy facilities, which is assumed to democratize decision making and the distribution of economic and social benefits of energy production [4].

While many renewables are highly compatible with a more decentralized energy system, where a larger variety of people own, manage, and benefit from energy infrastructure, technologies in themselves are neither inclusive nor beneficial. They are mediated by enabling or constraining policy environments and bureaucratic power structures, prevailing gender norms, power relations, and community structures [5]. Likewise, while the idea of ECs includes participative decision-making [6] and community ownership [7], research indicates that participation in ‘desirable’ energy activities and energy decision-making is influenced by social and economic factors, including gender, economic status, and homeownership [8, 9]. This article aims to contribute to this line of inquiry by exploring how and under which conditions energy communities allow for broader participation in the energy system. Previous research points at a paradox in this respect: although women have more significant environmental concerns than men [10, 11], ECs tend to be dominated by men [12, 13]. Some initiatives work towards the inclusion of more women in the renewable energy industry, like the *Women in Solar Initiative* [14]. There are indications that women can inspire other women to engage in energy-related activities [15, 16]. Nevertheless, relationships between gender and energy systems have not received significant attention from researchers [8]. Thus, more specifically, the paper investigates participation in solar energy communities in Sweden, focusing on the link between gender and decision-making.

In this regard, our contribution to the literature is twofold. A growing body of research grapples with disparities in engagement with residential solar PV and how they are related to demographic differences [17–20]. This paper examines how gender, as a more specific condition, influences the extent to which parties can or cannot engage with *collective* solar ownership models. Furthermore, the bulk of the literature on gender, energy, and climate change addresses women and their needs for greater energy access in the ‘Global South’^1^ [21, 22]. While these are indeed pressing issues to be researched, there is a persistent gap in the literature addressing gender, engagement with renewable energy, and leadership in the Global North [5]. In the seminal contribution *Gender and energy: is there a Northern perspective?*, Clancy and Roehr [23] argue that energy is seen as gender-neutral in a Northern context and that women and men are regarded as equal in their uses of and views about energy. Consequently, gender has been mainly made invisible in energy politics and policy in the Global North [24–28]. By highlighting the importance of gender for engagement with solar energy communities, this paper goes some way toward remedying this gap.

## Theory: gender, participation and decision-making

This research starts with the premise that energy cannot be fully understood without considering gender practices and cultural norms about gender [28]. We understand gender as roles and differences between men and women that are socially constructed. These roles are made from practices that are repeated and negotiated [29]. Gender is in this paper investigated from the perspective of women as decision-makers in energy communities. While we address gender in binary terms, due to the type of data we have been able to gather (see more in section ‘Data collection and analysis’), we wish to highlight that this does not reflect our perspective on the complexity and multiplicity of genders. We acknowledge that this is a limitation of the study, and we point to the eloquent problematization of how gender is addressed in energy studies by Fathallah and Pyakurel [30]. In line with practice-oriented research, practice is seen as the capacity to contribute to a world which is socially and culturally structured and constantly reconstituted by the activities of its participants [31]. Practice embodies the generative tension between not questioning the situatedness of one’s identity and actions, with the critical potential of being different and doing differently. Thus, we take on a critical approach that places gender and power relations central to the analysis [22] as we understand these as easily taken for granted by actors, but also mutable through practice.

To examine how gender affects participation in ECs, we draw on the work of scholars positioned at the gender-energy nexus, who critically examine power discrepancies and negotiations. Participation is herein understood in line with Frasier’s notion of the parity of participation, implying that just arrangements are those that enable all members of society to interact as peers [32]. We have found great support in the energy justice literature, highlighting the importance of broader participation in decision-making concerning energy access and the energy system’s design and need for restructuring [22, 33, 34]. The energy justice literature focuses on three dimensions: recognition, procedure, and distribution [22, 33, 35, 36]. The recognition dimension implies studying how women’s concerns, perspectives, arguments, and values are recognised. Indication of lack of recognition in our context would be active or passive marginalization or exclusion of women, and attribution of false values or opinions to women [37]. Procedural justice concerns how women are included formally; if they are heard and have a fair and real influence on the outcomes [33]. Distributive justice concerns the distribution of benefits, for example, if women can be seen as fairly impacted in terms of how compensation is designed [37]. The emergent body of research on the gender-energy nexus relates to the three dimension of justice and highlights the different social roles and positions that women and men occupy. Women have lower social status than men in most societies, which has brought inequitable workloads, fewer opportunities, resources, and assets ([22], p. 235). These different social positions affect the extent of their participation and decision-making in energy-related issues. Building on the findings of Clancy and Roehr [23], Standal et al. affirm that there is an established discourse in energy research and policy, which frames energy as gender-neutral [36]. By taking on a justice perspective focusing the relationship between the gendered aspects of social positions and decision-making in ECs, we aim to problematize this view on gender neutrality.

Decision-making in this article is referred to in the context of management positions of ECs. In line with procedural justice, we will consider if women in the decision-making process have (i) access, (ii) capacity, and (iii) the opportunity to influence developments [38]. We view decision-making as embedded and shaped by the cultural and organizational settings, wherein it takes place. Our perspective also resonates with Tjørring’s findings, who writes that decision-making is a process that plays out over time and through a variety of interactions [28]. Broader participation in decision-making for women or other marginalized groups is essential as it has the potential to enable them to act as role models for others and affect attitudes on gender in the sector [38]. We focus on the experiences as reported by board members.

We first examine *access* to decision-making processes. The literature on ECs affirms their potential for including those who for various reasons cannot invest in their own solar system, for example, because they do not have access to any suitable property or can afford to pay for an entire installation [39, 40], which also resonates with matters of distribution of resources and benefits. Research shows that by focusing on local involvement and cooperative values, ECs can involve groups other than those who traditionally invest in renewable electricity, e.g., women [16]. People interested in the social benefits of local, renewable electricity generation [41] can be reached. There is also support in the literature that women prefer to work in cooperatives, where through collaborative effort, they can overcome challenges, such as lack of technical knowledge [42]. These views on inclusiveness held by academics, and as we will show, board members alike, can risk creating a false recognition of justice and reproducing assumptions that citizens from different social groups are equally likely to participate in associational life [43].

When we discuss the *capacity* to participate we take into consideration that civic participation and technical knowledge possession are gendered. Boje et al. find that the gender difference in volunteering is higher in the Scandinavian countries than elsewhere in Europe [44]. They argue that the Scandinavian voluntary sector emphasizes sport, recreation, and political organizations, which are dominated by men in terms of both membership and leadership [44]. In contrast, women dominate among the volunteers in the welfare fields [44]. In terms of technical knowledge, the sphere of energy and energy systems development are often associated with male roles in engineering and technology, partly perpetuated by the ongoing gender gap in science, technology, engineering, and mathematics [21]. The labour market is gender-segregated all across the world, with women being underrepresented in STEM (science, technology, engineering, mathematics) and men in HEED (Health care, Elementary Education, and the Domestic spheres) fields [45, 46]. Concerning energy communities specifically, they tend to highlight that no technical knowledge is required for participation, but studies from, for example, Germany show that men (and people with high income and a university degree) are overrepresented in renewable energy initiatives [12, 13].

The final aspect of decision-making is related to *opportunity*. We will analyse this in a procedural justice perspective considering the opportunity to have formal access to positions of authority in ECs. Gender reduces women’s possibilities to participate as decision-makers in energy issues, as women are not equally represented in the energy sector’s managerial positions [23]. As this is the sector, where many of the board members are active in, the gendered patterns of staffing are reflected in the ECs. Research on Swedish energy companies’ boards confirms that men are overrepresented, with 72% [25]. Clancy and Feenstra refer to similar findings by IRENA on women’s engagement in renewable energy, wherein women make up 46% of the administrative posts, 28% of the technical staff, and 32% of senior management posts ([42], p. 25). Interviews with energy companies in Germany, Spain, and Sweden confirmed that gender equality efforts within decision-making are weak or non-existent [47]. While women’s role in pushing energy transitions forwards has been acknowledged [8, 33], this is far from the norm, as their visions and solutions are often disregarded [35].

## Methodology

### Study design and context

The research presented in this paper is a result of a qualitative approach to data gathering and analysis. We studied 11 solar energy communities throughout Sweden. Sweden is an interesting case, because its strong economic development in the twentieth century led to a relatively early development of nuclear power and extensive state involvement, which meant cheap and non-fossil electricity production [48]. The 1902 *Electricity Act*, maintained until the 1990s, stipulated electricity production by major power companies and its distribution by municipally owned companies [49]. These conditions have played into the inertia and low innovation rates of the energy market [48]. The electricity market was deregulated in 1996, implying that electricity production and sale were separated from the transmission, enabling competition [50]. Prosumers started producing wind and solar energy in 1997, and their existence has been supported by legislation on the sale of electricity back to the grid, state subsidies, and tax reductions [50]. The Swedish population has predominantly positive opinions on PV technology, with about 80% of the population stating that efforts towards implementation should increase [51]. However, PVs are still represented by only less than 1% of the total electricity production [52]. A capital subsidy for PV installations was introduced in 2009. The percentage of which and amount allocated has fluctuated throughout the years, but it ended in 2020, with a green tax deduction taking its place [52]. The Swedish PV market is dominated by customers who buy and own the PV systems, but large systems located away from end-users are becoming more common [52]. Nevertheless, interlocutors expressed dismay that cooperative owned solar energy production is not the subject of the micro-production tax reduction scheme, a possibility being investigated by the government since 2016 [53].

### Data collection and analysis

Data collection was done in two steps. First, in mid-august 2020 we mapped the existing solar energy communities in Sweden. This was done through an internet search using Google’s search engine and the search strings ‘solkooperativ’ (Eng: solar cooperative) and ‘solel, förening, andel’ (Eng: solar energy, association, share). The results were compared with previous research on energy communities in Sweden [54]. The resulting list contained ten active solar energy communities (organized as economic associations), two more in the making, and one housing association. All of the associations are run by a board of members. Most of them have relatively open membership requirements, although three of them require that members buy their electricity from a particular electricity retail company. The housing association involves ownership of an apartment in a specific area. All solar communities re-invest profits in new solar parks or refund earnings to their members by reducing their electricity bills. The associations also sell shares to companies, which vary between 2 and 10% of their members. It is noteworthy that these organizations have very poor digital visibility, which made locating them a challenge.

Second, we gathered data about the different communities and their members. Our primary method was semi-structured interviews. We have conducted 13 interviews with board members from 11 economic associations and six with the housing association’s board members. The gender composition of interviewees from the economic association was seven women and six men, and five men and one woman from the housing association. We employed the snowball method by asking our interlocutors about other board members to contact. Our approach and proximity to the interlocutors have been affected by the COVID-19 pandemic, forcing us to digitalize our interactions. Therefore, the interviews were conducted over Zoom, telephone, and Teams with a duration of 30 min to an hour. The questions were structured in themes, beginning with their professional background and experience with solar energy communities, continuing with questions about who partakes in these communities, what the enablers/difficulties might be for different groups, and ending in discussing the general and practical challenges of running such organizations. The interviews were transcribed and analysed manually in a qualitative content analysis. The subjects were promised anonymity, which is why we do not present any information that could disclose their identities.

It was rather difficult to obtain secondary data on the solar communities, since they have poorly updated webpages or even rely on energy companies’ subpages. Moreover, there were GDPR challenges in accessing information about member composition and accessing individual members of the ECs. Therefore, we were dependent on the board members cooperation to make their member lists GDPR-friendly. Five of the solar communities decided to help us, but the data we got varied in terms of specificity, from a percentage summary about gender and age to lists containing all members’ first name and birth year. The ECs had between eight and over 300 members. In the smallest EC, members were locals, but the other ECs had local and national members (or shareholders). The share of women among the members was 23–48%, with an average of 37%. Three of the ECs estimated that the average member age was 50–55 and 60, respectively. These ages are similar to the numbers reported in a previous study of an energy community in Sweden, where the average age of members was 58 years [54]. Interlocutors suggested that this was an age group with the time and money to invest. Other groups might not be able to invest due to demanding jobs or dependents to tend to. This agrees with Clancy and Feenstra’s study, which shows that housework is unevenly distributed between women and men in the EU; in the period of 2005–2015, almost one working woman in two spent 1 h a day on caring activities, compared with one out of three working men [42]. The 10 ECs we examined have 70 board members altogether, whereof 62% are men. The housing association’s board consists of 50% women and 50% men. It is worth mentioning that two ECs have a majority of women board members (57% and 60%, respectively). All chairpersons were men.

None of the associations monitors their members’ ethnicity, but based on interview statements, the members are predominantly ethnic Swedes. The challenges with accessing data and interlocutors during the pandemic have also impacted the kind of analysis we have been able to make. An intersectional perspective, considering factors, such as class, sexuality, education and disability, would have significantly added to this article. We argue that it is essential for future qualitative research to take these factors into consideration and examine how they overlap and affect renewable energy engagement. Viewing the membership statistics and the composition and representativity of the boards indicates a gendered pattern of decision-making, as we will discuss in the following.

## Results and discussion: women as decision-makers in ECs

We now go on to present our results and analysis. They are structured into three subsections, wherein we address access, capacity, and opportunity to participate in ECs. From the vantage point of access, we discuss the perceived attractiveness of solar energy and the inclusiveness of ECs as organizational forms. We will argue that egalitarian views have paradoxically led to a lack of attention for diversity. We then address capacity concerning support from energy companies to show how organizational embeddedness and associational experience can contribute to the longevity of an initiative and affect the types of engaged actors. Through opportunity, we examine women’s board member experiences in ECs. We show that women’s opportunities to engage in the boards are positively affected by other’s women as role-models and peers. We also show that the boards of ECs replicate gendered recruitment patterns present in the energy sector and STEM.

### Access: accessible energy sources and organizational forms

#### The attraction of solar energy

The following section is about the perceived attraction of solar energy and the expectations board members have in increasing the participation rates, particularly on behalf of women. We encountered reoccurring themes, some of these affirming stereotypes of women and female behaviour. According to Eagly and Steffen, stereotypes are related to social structures and the distribution of social roles [55]. They both represent and distort reality, and according to these authors, will remain as long as there are unequal distributions of social roles [55]. Thus, we have chosen not to discard these themes but to analyse them as an aspect of recognition, and to see what they indicate about women’s engagement with renewable energy initiatives.

The most common theme in terms of the appeal of solar energy for women was their perceived closeness to nature and family orientation. ‘It feels like, to put it quite unscientifically, that the sun as an energy source appeals to women quite a lot’ (Interview 2b), we were told by an interlocutor from one of Sweden’s first ECs. Or as another interlocutor put it: ‘Women tend more to the children and grandchildren, and what (the kind of future) they are going to inherit’ (Interview 1b). Women were attributed with values, such as vulnerability to climate change, closeness to nature, virtuousness, and with roles as mothers and protectors. These have also been strategically invoked in ‘Western’ environmental activism, as well as echoed in much of the literature about gender and climate change [8]. Women environmentalists speak of protecting children, ensuring a future for coming generations, preserving the home and family life, and maintaining health and quality of life for people in their communities ([8], p. 4). Some international findings confirm women’s stronger environmental attitudes and behaviours than men [11]. For example, research from the US shows that men tend to be less likely to be concerned about environmental harms and less likely to engage in pro-environmental actions in daily life than women [33]. A tendency to be disengaged with environmental challenges stems from the desire to protect a masculine identity and the social privilege it affords [33]. As we see, the links between women, nature, and family orientation are recognised widely both in research, activism, and practitioner discourses. Such discourses attribute false values on women and contribute to naturalizing women as stewards of nature, placing upon them the burdens of adjusting their behaviours to tend to their families’ social needs and to their communities’ environmental needs.

Another reoccurring theme was women’s perceived high-risk aversion and low trust in technology,

We have worked here at the energy company with gas sales since the 1980s, with the gas network. With gas, there is an apparent division. Women do not want gas at home, but it is older men, engineers who think it is great with gas. You have such high faith in the technology, and you know exactly how it works. If you are not technically interested, you have more confidence in solar energy because it’s risk-free... It appeals to women more because it’s more about the species’ survival, and you can feel it in your heart, like, solar energy feels safe and right in every possible way. (Interview 1b)

This interlocutor works in the energy sector, and it came through during the interview that they had reflected upon women’s engagement with different energy sources and different technologies over a long period of time. These perceptions are consistent with findings from the US and Germany, which confirm that women are more concerned than are men about a wide range of risks [33, 43, 55]. According to Swedish findings, women are more concerned than men about environmental risks [10]. White men stand apart from other groups when it comes to risk perception. The explanation for such a position is related to procedural justice, that white men tend to be more often in a position of power than other groups, and this privilege allows them to perceive the world as less dangerous [47]. A study from the US finds that women tend to trust science and technology less than men, and that trust in science and technology is negatively related to environmental concerns [56]. Women are not only more concerned about environmental risks: they are also more prepared to act upon them [56]. More Swedish women than men experience that they can act to curb climate change [47]. They are also prepared to reduce their carbon dioxide emissions [47]. Thus, in theory, women’s lower risk tolerance and lower trust in technology positions solar energy as a desirable choice.

We have herein presented the perceptions held by interlocutors in terms of how gender affects participation. Interestingly, solar energy was recognised as an appealing choice for women. It was perceived as resonating with their environmental concerns, their family orientation, and their aversion to risk and low trust in technology. While these themes reoccur in activist, research, and practitioner accounts, they are also problematic from a justice perspective. They can leave unjust power relationships unexamined and could distribute unfair expectations upon women to act as stewards of nature.

#### ECs as inclusive organizational forms

This section is about the member composition of ECs and the board members’ lack of recognition of insufficient diversity. One of the energy communities studied was founded during the 1980s as a housing association focusing on ecologically well-thought-out and energy-efficient housing. The community was built on an aspiration to live according to a long-term sustainable strategy, where the design and residents’ everyday lives had to consider environmental impacts and encourage social activities. During the interviews, it became clear that this EC reflected the social motivations for engaging described in earlier research [57]. The community was organised in different working groups running everyday activities. As described by one of the interviewees, these working groups were, however, gendered: ‘The movie group is only men; the workshop group is only men… Also, the energy group consists of men. That is, of course, a pity’ (Interview 4a).

The gender divide was in this sense reflected by the organization, despite their gender-balanced board. During interviews, interlocutors of the other ECs stated that they perceived their solar ECs as quite inclusive. They were not selective about who the members were, or as one interlocutor put it, ‘we are not interested in where the person comes from and what they look like’ (Interview 1b). On the webpage of this EC it is highlighted that the opportunity is there for ‘people from the entire country’, and they do not require potential members to be customers of the energy company they worked with. ECs were also perceived as less capital intensive than household installation of solar panels,^2^ they do not require installations on the housing units of the members, they do not require knowledge on the technology on behalf of the members, and shareholder engagement is not time demanding, as most ECs meet with members once a year. There are likewise no requirements on the extent of members’ engagement, beyond the purchase of a share to be enrolled. There was a sense of accomplishment expressed by multiple interlocutors on the point of their communities being inclusive, particularly of women, as the following answer to a question about the relative distribution of women and men among the members shows: **‘**I have to look a little carefully (at the member lists), but I think you would be surprised how many women there are’ (Interview 2b). Men tended to speak about the inclusion of women more often, which could be associated with social desirability issues, which implies that they have provided a more socially acceptable answer in a country, where gender equality is a dominant discourse.

None of the interlocutors engaged in a discussion on the inclusion of people of a varied ethnic origin or young people at great length. Language skills or lack of economic capital were briefly referred to as the absence of the former or the latter group. In terms of most of the members being fifty and older, interlocutors stated that this generation would like to make up for the unsustainable lifestyles they might have led and invest into their children and grandchildren’s future. As we noted in section ‘Data collection and analysis’, this is an age group that has the time and money to invest. One interlocutor from the housing association reasoned like this when asked if gender equality is an important issue for the community:

I cannot imagine that anyone actively or consciously would oppose or reject equality…but no I have not experienced this as a problem and this is probably easy to say for a white man. Still, no, I have not experienced any issues with equal treatment. (Interview 5a)

From the gathered data, it can be observed that the studied ECs had egalitarian ideals. At its outset, the housing association aspired to stimulate social and inclusive lifestyles. The other ECs perceived their organizations as inclusive due to the little that they demanded of their members. The most significant investment was the decision to come on board and pay the fee. While energy communities can, in theory, make it possible for more groups to invest in solar production, it does not always look like that in practice [12]. Not all citizens can participate in associational life [43]. The following interlocutor is making a point that there are different kinds of social activities that women and men engage in:

I think it is often men (who join ECs); you can also see that it is usually men on the boards in tenant-owner associations. This is because women have so many other social networks. You hang out with your girlfriends and have different groupings. But men are pretty bad at that general social stuff. So then it is appropriate for them to join associations because they get a network. Especially if there is something about the technology involved, and it is with solar energy. That’s why I think it’s men who get involved. Slightly older men who may have the time to… Men need something to do when they retire. Women often have it. (Interview 3b)

This interlocutor was from an EC with around 20 members from the local area, where most of them knew each other. During the interview, they confirmed that men might dispose of more free time than women due to the differences in the amount of care labour performed (tending to family members, the home and cooking). Civic associations are, furthermore, not an organizational form that appeals or is available to people of all socioeconomic or cultural backgrounds. Scandinavian countries have high rates of civic engagement when compared to other EU countries. However, men still have a slightly higher probability of doing voluntary work than women, and participation rates are low for ethnic minority groups, poor people, etc. [44, 58, 59]. The research on social capital in Europe by van Oorschot, Arts, and Gelissen confirms this point [59]. These scholars argue that European women are more involved in informal helping and neighbourhood activities, while men take the lead in voluntary organizations. Studies on women’s involvement in citizen participation schemes in renewable electricity production in Germany indicate similar findings, as women’s participation seems to be lower than those of men in general. On average, 22% of the owners are women, and 75% are men [43]. The data we gathered with the Swedish ECs also indicates that women are a minority both as members and managers. Therefore, the question is if community energy can provide services and engage with communities broadly, or if they are likely to concentrate on well-resourced groups [60] is also relevant for the cases presented herein.

When it was possible to choose between different working groups in an EC, such as the housing association, the energy group was occupied by men. At the same time, the women engaged in other groups not related to energy. Energy was seen as something technical, as knowledge that men brought with them before the EC membership (Interview 6a). When there is no physical intervention of the members’ households, electricity is still symbolically tied into the household, which is the most gendered spheres across societies [61]. As such, for better or for worse, owning a share in a solar EC does not affect the members’ everyday lives. Neither does it challenge the social relationships, divisions of labour, and power structures they engage in. Abstract ideas of equal opportunity can mask the existence of deep, structural injustices [33, 36]. According to Johnson et al., renewable energy projects cannot achieve gender and social equity, as energy interventions do not automatically tackle the structural dynamics embedded within socio-cultural and socioeconomic contexts ([5], p. 2). This seems to be confirmed in our cases.

### Capacity: support from energy companies and its effect on diversity

The following section will examine the effect of the support offered by energy companies. While we confirm previous research findings that position support is essential for the longevity of the ECs, we argue that it affects the lack of diversity of their managerial composition. Seven out of the 11 ECs have been started by or in cooperation with energy companies, with several interim board members from these companies. The data gathered confirmed findings on the topic that champions in the companies interested in renewable energy took the initiative to start up [62]. Ideally, local citizens are the driving force in each step of realizing a renewable energy community: planning, mobilization of resources, and its operative implementation [43, 63]. However, an institutional story of origin is common in Sweden. As the electricity market is centralized, there is already a high share of renewable energy in the system, and the low electricity prices leave little room for incentives by grassroots [62]. Based on findings in Sweden, citizen involvement, Magnusson and Palm argue, is a niche phenomenon, dependent on a community’s access to capital, technical knowledge, and institutional settings [62]. There are few case studies of renewable energy communities in poor communities, as they experienced challenges in obtaining resources, such as money, material, knowledge, and time [62]. The following quote from and EC that works closely with an energy company and has a board member that is an employee of that company confirms the view that institutional support is necessary to manage the aspects that volunteers do not have time or expertise for:

He does this (board member duty) partly during his working hours because the company is also a partner in this. If he drops out, then it would be difficult without the skills (he has). We are all doing this non-profit. And… he has that competence (which is required). The rest of us are pretty exchangeable on the board. Some of us are, say, good at social media, but many can do that. (Interview 5b)

We can see a facility coming from the backing of energy companies, as in the case above; for example, a technical expert on the board could be paid for their time. Nevertheless, there was the issue of person dependency, as the person held knowledge and skills deemed essential. If the company restructured this person’s work assignments, it would dramatically change the situation of the EC. Interlocutors also told us that it was not easy to build a board as not many were interested in becoming board members. Board members from ECs who collaborated with energy companies stated that it was difficult to find others who would dedicate time without paying for it (Interview 1b). The boards’ missing spots are filled through the companies or their partners investing in the ECs (Interview 13b). When the initiative did come from the grassroots, the engagement with energy companies was not only perceived as benefiting the ECs, but also the companies:

For them, it became a good advertisement that they create electricity in this way. And we got good financial conditions because we needed to pay our debts quickly. For both liquidity and solvency in the association. And they may not have had the strength to do this. We have personal relationships with people who could be shareholders. We were this soft power. And they had the muscles, and it became a good combo. (Interview 3b)

This grassroots EC was initiated by a group that had a history of working together on various initiatives. While they only had 20 members at the time the research was conducted, the interviewee stated that they all had different competencies and different technical knowledge levels. Most importantly, they all had contacts who could supply them with technical expertise when necessary. Their shared experiences made it possible to mobilize their social capital and other resources efficiently. They acknowledged the energy company’s role in supporting them technically and economically, but they also perceived the relationships as a symmetric exchange, wherein they supplied the social relationships. The networks they had established—and having started the EC due to their personal engagement—brought them a feeling of ownership and independence that increased their decision-making capacity.

As we can see from the cases presented in this section, the advantages of creating and EC with an energy company’s backing were multiple. They were able to draw on former experiences of organization and business models; there was contact with customers who could be potential members, expert knowledge, and the capacity to put the infrastructure into place. Smaller grassroots initiatives also needed to find a fruitful collaboration with energy companies and maintain an elaborate network of contacts that could be mobilized and maintained a certain level of their independence. The ECs founded by energy companies relied on their staff and institutional networks for filling the board positions. The grassroots initiatives, on the other hand, had their reliance on members who have cooperative experience. This can be tied into Wirth’s argument on the circularity of familiarity with cooperative forms of ownership, leading to homogeneity among groups of actors and lacking heterogeneity conditions [64]. As shown in the former section, it is still men who still have a slightly higher probability of doing voluntary work [44]. From these perspectives, it can be gathered that while organizational embeddedness and associational experience contribute to the longevity of an initiative, they can also affect the types of actors that are engaged. In a procedural justice perspective, actors will have a privileged position based on their technical knowledge and their already acquired capacity to navigate organizations and mobilize resources.

### Opportunity: women as board members

This section will explore how women experienced procedural justice and their possibility to participate in the boards, and the barriers and enablers they encountered. Formal access to management positions in ECs is a condition to have the opportunity to engage in the decision-making process and accrue decision-making power [38]. While the numbers reporting the gender ratio within EC’s in Sweden indicate that the Swedish ECs engage relatively higher percentages of women than their counterparts in, for example, the aforementioned cases in Germany, solely focusing on the percentage of members risks diverting the gaze away from issues of power structures and leadership. None of the interviewees reported unpleasant experiences on boards due to their gender. The interviewed women reported that their experiences were positively affected by being surrounded by other women who played a similar role:

There were several of us, Lisa^3^ was also there… So, we were two women and three men or something like that, or maybe they (the men) were four, I do not know, so it did not feel so strange. And then if you have been (working), like me, in the real estate industry and the construction industry, then you are as used to it, to the narrative, or what to call it, of (working with) middle-aged white men... There was no difference, but it was fun that Lisa was so driven and then Amanda, my boss (was there), so there were still women there. (Interview 13b)

This interlocutor was a board member in an EC initiated by an energy company. While this interlocutor experienced her time on the board as friction-free, one must consider her employment history in male-dominated sectors. This background enabled her to navigate new spaces and practices, wherein women were a minority. Fraune [43] draws a parallel between renewable energy production and German sports organizations. Women are underrepresented in executive bodies of sports organizations, since a precondition is a long and continued commitment to sport and sports organizations. In relation to renewable energy production, experience in either business or technology, which many women might lack due to the aforementioned gender segregation of STEM, might be a precondition for taking part in executive boards. The interlocutors’ statement also confirms that women can inspire other women to start engaging in energy-related activities [15, 16]. The research about women in sport organizations also indicates the importance of female role models [43], and role models enhance girls and women’s attitudes towards STEM as a possible career choice [42]. Research on women’s professional networking in the sphere of renewable energy in the USA and Canada confirms that mentoring is critical to women’s professional development. Mentoring and networking are among the most critical factors leading to career success for those employed in the U.S. solar energy industry [65].

While the ECs were represented as welcoming of women, both as members and on boards, one interlocutor did indicate that in the energy branch in general, being a woman and a non-white person could pose obstacles to one’s professional identity:

So, you are often questioned when in groups, if you do not know the people. I am questioned in the circle I am in because it is still unusual for a female, non-white person to be involved. But it is not a problem, as soon as you have explained who you are and what you have done. But you do have to explain yourself, unlike if you had been a white man. It is not a matter of course that I enter a room and that everyone understands what I can do. (Interview 12b)

The interlocutor quoted herein underlines that their positionality affects the extent of their everyday professional interactions in the energy sector. ECs tend to score better in terms of energy justice, as they can, in theory, provide joint ownership, decision-making, and access to the profits generated. However, research does indicate that problematic aspects can arise, such as in the differences between involved individuals and how they engage in participation [33]. Other interlocutors have not referred to these kinds of interactional issues with regards to the ECs.

We now discuss the gendered structure of decision making is and how the power relationships look in the boards. Findings form the energy sector in Europe and Sweden confirm that women are a minority [23, 25, 27]. Thus, the board compositions reflect general patterns in this sector:

They are engineers. We laugh at that too. I am not, but many are. On the board, we are two environmental scientists. Yes, it is engineer-driven. And you can understand that, they understand electricity ... (laughs). (Interview 5b)

The board that this interlocutor was a part of had experts from engineering. This was particularly the case with the ECs initiated by energy companies. The gender segregation of the labour market and women’s underrepresentation in STEM thus gains special importance for the matter [45]. Issues of women’s leadership and inequality are particularly salient in STEM-related fields and activities. As forementioned, research has here confirmed the importance of role models and mentorship for women’s inclusion, particularly in leadership [65, 66]. Women can inspire and engage more women, but for this to happen, there need to be structural preconditions in place, such as gender policies for the boards. Johnson et al. argue that not taking gender into account can benefit the groups that are already in a more privileged position, such as men, who are more often recruited in the system of energy supply [5]. Thus, if there is no greater awareness of the gendered patterns of recruitment and volunteering in the energy sector, the ECs risk replicating the sector’s inequalities. Women and underrepresented groups are essential enablers of change. They need to have real agency in participation, recognition, and decision making [21] in energy system transition and to be able to reach the goals in EU’s Clean Energy for all package.

From this section, it can be gathered that women’s opportunities to engage in EC’s boards are positively affected by other’s women functioning as role-models and peers. Our interview data nevertheless indicates that the energy sector has problems when it comes to procedural justice and especially in relation to inclusion and diversity. Our findings confirm that the boards of ECs replicate gendered patterns in the energy sector and STEM more generally, benefiting men who are the primary group to be recruited.

## Conclusion and implications for further research

The purpose of this paper was to investigate participation in solar energy communities, with a focus on the link between justice and the gender-energy nexus, by means of a qualitative study of 11 solar energy communities and one housing association in Sweden.

The study revealed that despite the relative potential for inclusion that ECs hold, most of the members were men. In a procedural perspective this unbalance became even more pronounced. The boards and management teams were dominated by men. Moreover, in the EC that had a specific energy group, only men participated in that group. Findings on engagement in energy production in Norway and the United Kingdom confirmed that there exists social differentiation along gender lines, with energy and technology being perceived as masculine domains, and thus continuing to impede women’s participation [67].

Nevertheless, the interviewed board members perceived the organizations as inclusive. They did not recognise any gender injustice and were of the opinion that all were welcome as members. Some emphasized that they had not experienced any problems with lack of inclusiveness or unjust treatment. They were uneasy with recognising any injustice in their EC and argued, among other things, that no knowledge of the technology was required and that the ECs demanded very little engagement and time from their members and, therefore, did not intrude on their everyday lives. This perceived inclusivity was framed within the limitations of the structural and gendered injustice of society in general, where women have lower status, fewer opportunities, resources [33, 35], as well as spend more time on performing care labour [42].

Several themes were recurring among interlocutors and were used to uphold a discourse on ECs as contributing to energy justice and as inclusive to anyone who wants to become a member. Most notably, gender and being a woman, was not perceived as an impediment to participation in ECs but rather as an enabler, since solar energy and solar PV technology were perceived as being especially appealing to and accessible for women. These themes were tied into power inequalities acknowledged in previous literature, such as women’s greater dependence on their environments, higher exposure to risk than white men, and the gendered segregation of STEM [33, 35, 45].

Taken together, these results indicate that energy communities raise justice concerns in terms of inequities concerning access, capacity, and opportunity to engage in decision-making in ECs. While similar findings have been reported in a few previous case studies [33, 35, 43], this further puts the European Commission’s ambition to use energy communities as a means to develop an inclusive, equal, and effective energy market into doubt. Even if energy communities, because of their organizational form, have the potential to promote a more equitable and just inclusion than traditional energy systems, it is clear that policymakers might need to take more direct action and adopt regulations and incentives to make the jointly owned solar energy generation accessible to more groups in society.

Tjørring writes that there are cultural associations between energy and masculinity and that women categorize doing something for the environment with nature [28]. All our interlocutors expressed that they value the environment, and they recognized the importance of sustainable living. A possible direction for the future for these associations is to emphasize the connection between energy and the environment, as a way to decouple energy as a man’s domain.

Some of the limitations of this study point to fruitful fields for further research. First, due to the pandemic restrictions, negotiations within a household concerning engagement in ECs could not be studied. As previous studies have shown that while women take an active role in decision making [56], they do not continue their engagement in organizational participation, because energy has been culturally associated as a man’s domain [28]. Further research could show if this explains the low participation rate in solar energy communities in Sweden and elsewhere. Second, an intersectional perspective on class, ethnicity, age and ability could be taken up by further research to uncover how these factors into renewable energy engagement.

While solely focusing on gender offers a limited view of the dynamics of participation in renewable energy projects, it is our position that integrating gender into the analysis will provide insights into possible measures to remedy limitations and accelerate the renewable energy transition.

## Data Availability

The data that supported the findings are generated through qualitative ethnographic research. It cannot be made available in consideration to research subjects’ privacy.

## Abbreviations

EC: Energy community
EU: European Union
HEED: Health care, Elementary Education, and the Domestic spheres
IRENA: International Renewable Energy Agency
PV: Photovoltaic
STEM: Science, technology, engineering and mathematics

## Footnotes

1 We agree with Clancy and Roehr [23] that Global South and North are problematic terms. Like these scholars, we use them as a shorthand to designate the industrialised countries belonging to the Organisation for Economic Cooperation and Development (OECD), and the Eastern European countries, known as the Accession countries.

2 The prices for purchasing a share in the ECs varied between 850 SEK and 15,000 SEK. A normal-sized villa system costs approx. SEK 11,500/kW (including ROT (Repairs, Conversion, Extension) deductions). For comparison, the annual average household disposable income for 2019 in Sweden was 489,900 SEK.

3 The names are pseudonyms.
